# Influence of Tire Rubber Particles Addition in Different Branching Degrees Polyethylene Matrix Composites on Physical and Structural Behavior

**DOI:** 10.3390/polym13193213

**Published:** 2021-09-22

**Authors:** Marc Marín-Genescà, Ramon Mujal Rosas, Jordi García Amorós, Lluis Massagues Vidal, Xavier Colom Fajula

**Affiliations:** 1 Mechanical Engineering Department, Escola Tècnica Superior d’Enginyeria Química, Universitat Rovira i Virgili (ETSEQ-URV), 43007 Tarragona, Spain; 2 Electrical Engineering Department, Escola d’Enginyeria de Terrassa, Universitat Politècnica de Catalunya (EET-UPC), 08222 Terrassa, Spain; ramon.mujal@upc.edu; 3 Electrical Engineering Department, Escola Tècnica Superior d’Enginyeria Química, Universitat Rovira i Virgili (ETSE-URV), 43007 Tarragona, Spain; jordi.garcia-amoros@urv.cat (J.G.A.); lluis.massagues@urv.cat (L.M.V.); 4 Chemical Engineering Department, Escola d’Enginyeria de Terrassa, Universitat Politècnica de Catalunya (EET-UPC), 08222 Terrassa, Spain; xavier.colom@upc.edu

**Keywords:** electrical properties, electrical modulus, composites, structural features, ground tire rubber, polyethylene, recycling

## Abstract

Waste from pneumatic wheels is one of the major environmental problems, and the scientific community is looking for methods to recycle this type of waste. In this paper, ground tire rubber particles (GTR) from disused tires have been mixed with samples of low-density polyethylene (LDPE) and high-density polyethylene (HDPE), and morphological tests have been performed using scanning electron microscopy (SEM), as well as the dynamic electric analysis (DEA) dielectric characterization technique using impedance spectroscopy. From this experience, how GTR reinforcement influences polyethylene and what influence GTR particles have on the branched polyethylene has been detected. For pure LDPE samples, a Debye-type dielectric behavior is observed with an imperfect semicircle, which depends on the temperature, as it shows differences for the samples at 30 °C and 120 °C, unlike the HDPE samples, which do not show such a trend. The behavior in samples with Debye behavior is like an almost perfect dipole and is due to the crystalline behavior of polyethylene at high temperature and without any reinforcement. These have been obtained evidence that for branched PE (LPDE) the Maxwell Wagner Sillars (MWS) effect is highly remarkable and that this happens due to the intrachain polarization effect combined with MWS. This means that the permittivity and conductivity at LDPE/50%GTR are high than LDPE/70%GTR. However, it does not always occur that way with HDPE composites in which HDPE/70%GTR has the highest values of permittivity and conductivity, due to the presence of conductive fraction (Carbon Black-30%) in the GTR particles and their dielectric behavior.

## 1. Introduction

Waste tires are a huge and global environmental problem. Tires are made of natural rubber and plastic, and they contribute to the pollution of our seas and environment. Fragments of degraded plastic from tires are invading the environment, including the seas. Between 1–1.4 trillion tires are produced and sold yearly around the world, increasing production each year of between 2–3% [1,2,3], these are relevant waste emissions that our planet cannot afford without a recycling strategy for these compounds, as they have a very high pollutant potential and are challenging to recycle. 

End-of-life tires (GTR) after their life cycle go largely to landfills, or energy recovery, or on the other hand are also to reused and mixed with other industrial polymers for use in various applications such as playgrounds (either with asphalt or other insulation or building materials). In the GTR valorization point of view, the option of energy recovery is the most common when managing tires once they have ended their useful life (45%) [4]. With the consequent problems of toxic emissions generated by the combustion of these wastes, other GTR treatment options are waste stocks (30%), reused (15%), and finally recycling with 10% from the total of the amount of end-of-life tires treatments after the life cycle of tires. 

Recent research works explore waste composite polymeric materials as potential materials for use in electrical insulation [5,6,7,8,9], generally for low insulation requirements. The present research is shown the structural-dielectric behavior from mixtures using one of the most used polymers in the industry such as polyethylene, in its most common varieties: high density (HDPE) and low density (LDPE).

High-density polyethylene is a thermoplastic polymer made up of repeating units of ethylene. If there is branching it is called branched polyethylene, low density, or LDPE. When there is no branching, it is called linear polyethylene, or HDPE. HDPE has a low level of branching, high intermolecular forces, and a high density (0.952 g/cm^3^). LDPE, introduce longer olefin comonomers, is called low-density branched polyethylene, or LDPE. At the electrical level, polyethylene is non-polar, since the most common groups such as CH and CC in their composition are not polar, which is why the hydrocarbons present in polyethylene polymers are non-polar [10,11]. This manuscript includes an analysis from low-density and high-density structures, within the same polymer (Polyethylene), and how the particles reinforcement (GTR particles) has affected the structural and dielectric levels is examined. Comparative studies of this type are unprecedented in the case of polyethylene, so the comparison presented in this manuscript helps to understand how it has affected the tire particles on two structures with different degrees of branched and density.

**Figure 1:**
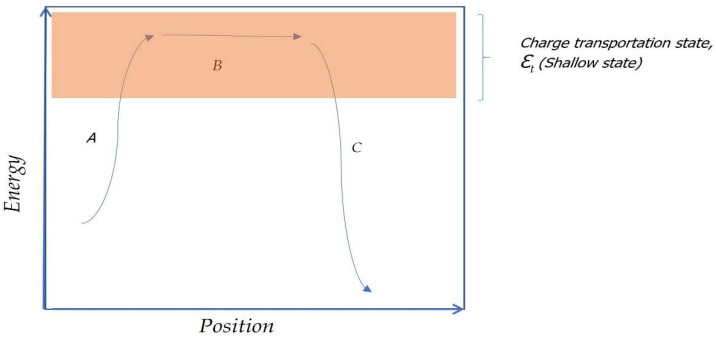
Charge carrier mechanisms: Hopping conduction processes for polymeric insulators.

The phenomenon of electrical conduction in insulating solids is explained by hopping transport mechanisms [12,13,14,15], where electrons jump from lower energy levels to higher ones. Thanks to the arrangement of holes in higher energy levels that allow excited electrons to move to charge transport zones located at higher energy levels, these transport mechanisms of charges explain the electrical conduction for insulating polymers such as polyethylene (although there are other phenomena of electrical conduction such as superconduction, and band transport mechanisms or tunneling for other types of materials). In polymeric insulators, the electrical conduction is governed by Hopping transportation processes mechanisms, in which the presence of excited electrons, as well as holes, causes a “jump” of excited electrons to holes or “vacant” electrons. Therefore the presence of excited electrons and holes is essential for this phenomenon to be reproduced, in the process of transport of charges the steps that can be glimpsed in Figure 1 take place. Thus a series of steps are described: (A) an electron or excited molecular dipole structure is transferred to transport energy states (ԑ_t_); (B) the transport of charges in this transport zone occurs, to energetically excited zones that can produce charge transport; and (C) at this point, the charges lose their energy state and can be trapped in less energetically excited areas and therefore with fewer transport possibilities [16,17]. Therefore a direct relationship appears between the energy state of the particle and its transport probabilities.

The charges transportation by polarization effects is explained by Debye’s theory [18,19,20], in which dependence of the polarizability (α) is assumed to be directly proportional to $\alpha_{0}$ (orientation static polarizability) and inversely proportional to the frequency of the applied field w and to the relaxation time т, the part of the polarizability due to the orientation depends on the frequency of the form which is shown in the following equation:

(1) $$\alpha (w)=\frac{\alpha_{0}}{1-iw\tau }$$

At the dielectric conductivity level, the results at moderate temperatures (30 °C) are analyzed, including the results of the conductivity with the dependence of the frequencies (from 10^−2^ to 3 × 10^6^ Hz), and the conductivity behavior is already described by Jonscher [21,22], where the conductivity is divided into an AC part (alternating current) and another DC part (direct current) of the conductivity, as described in the next equation:

*σ_ac_* = *σ_dc_* + *Aω^s^* (2)

In this expression (1) σ_dc_ is observed, which is the frequency-independent conductivity parameter (conductivity DC part), and the frequency-dependent parameter σ_ac_, in addition to the angular frequency (w) itself and structural parameters *A* and *s* which take ranges of values between 0 and 1 and which may depend on the temperature, the amount of GTR reinforcement, and the material of the matrix itself.

Physical–dielectric behavior has been characterized for two reasons: at the physical level, the changes that produce the GTR amounts in different branching degrees, especially in permittivity and conductivity to understand the dielectric changes studied and to analyze possible dielectric applications from the mentioned composites, due to the field of dielectric composites is a research field with many recycling possibilities to analyze.

## 2. Methods and Materials

In this research, impedance spectroscopy is applied to the study of dielectric behavior and the study of composite material structures. Dielectric spectroscopy (also called impedance spectroscopy) measures the dielectric properties of a medium as a function of frequency. The method is based on the interaction of an external field with an electric dipole moment of the sample, expressed as the permittivity. The analyses have been carried out with an impedance spectroscopy machine, namely the DEA test (dynamic electric analysis) BDS40 unit Novocontrol, with a Novotherm temperature sensor incorporated. For this test, a piece of a test piece, as large as a token, with dimensions of 2.5 cm in diameter and 0.1 mm thick, is placed between two electrodes in contact with the sample. When the DEA test is activated, an alternating current between the two electrodes, applying a field voltage of 1 Volt, is produced. Two responses result from this current, namely a resistive and a reactive current. The phase shift between these two currents calculates the electrical parameters, namely real permittivity, imaginary permittivity, and conductivity. The analyses have been carried out in a range of different frequencies (1 × 10^−2^ to 3 × 10^6^ Hz) and temperatures. Regarding the structure analysis, scanning electron microscopy (SEM) was used for internal structure analysis from fracture surface micrographs of the broken compound’s specimens. The effects of this filler material on the matrix can be analyzed by observing the fracture surface of the polymer with the reinforcing particles. A JEOL 5610 microscope was used, and the samples were previously coated with a thin layer of gold to increase conductivity. The samples were photographed at 180–400 magnifications.

### Sample Manufacturing

As can be seen in the comparative Table 1 of HDPE-LDPE values, some of the most important physical properties for the tested materials are presented, in general, it can be observed that the characteristics present great similarities at the level of physical-mechanical behavior.

The HDPE used [ALCUDIA 4810-B] was supplied by Repsol YPF company, and it is high-density, linear, granulated polyethylene. As for used LDPE, branched linear low-density polyethylene, [LDPE-superhexene1 Exelene], and it is a granulated LDPE from Montachem International company. About the perform samples phase, the mixture weighted with 0–5–10–20–40–50 and 70% of GTR particles amount is processed in plastograph or laminating machine, namely the Plastograph EC-Brabender company (Duisburg, Germany). After this process phase of 10 min, the next phase is a press roller machine with the obtained mixture from plastograph. After the press phase, the sample is cooled and, finally, the sample is cut at sizes according to the standards and test sizes. In the DEA test the sizes are near than 0.1 mm of thickness and a diameter of 2.5 cm. GTR with no metals and textile has been supplied by Pneumatics Maials and micronized and separated by sieving in a research laboratory in a <200 microns of particle diameter.

## 3. Results and Discussion

### 3.1. Internal Structure—Micrographs Analysis

**Figure 2:**
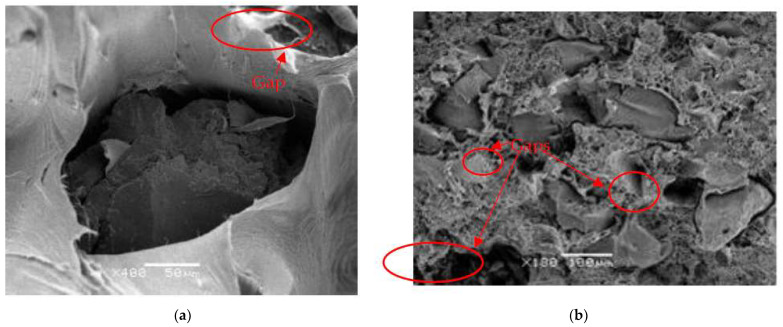
Scanning electron microscopy (SEM) micrographs of (**a**) LDPE/20%GTR compounds at 400 magnification and (**b**) HDPE/20%GTR-compounds at 180 magnifications.

At the microstructure level from the visualization of the samples with electron microscopy, using the SEM equipment JEOL (Tokyo, Japan) in Figure 2, in 400 and 180 magnifications, respectively, the previously sputtering process has been applied, coating the broken specimens in a 20-nanometer layer of gold. Two differentiated phases are observed in the analyzed composites. On the one hand, the polyethylene matrix (LDPE-HDPE, respectively), according to Figure 2, and on the other hand, the reinforcement of tire particles. Therefore, the integration of the reinforcement in the matrix appears as a differentiated phase, with little integration in the polymer matrix as spaces between the particles and the reinforcement are observed, results that are common in these types of composites [23]. On the other hand, a highlight is a homogeneity in the reinforcement of the particles in the composite, since as seen in Figure 2b this reinforcement appears with a good dispersion throughout the sample, which is important to verify that there has been no accumulation of particles throughout the manufacturing process of the samples. Therefore, it is confirmed from the images shown that there is low internal cohesion between the two phases of the composite and good internal dispersion of the reinforcement in the matrix of the composite, which certainly indicates that the samples have been made correctly.

### 3.2. Conductivity Analysis

**Figure 3:**
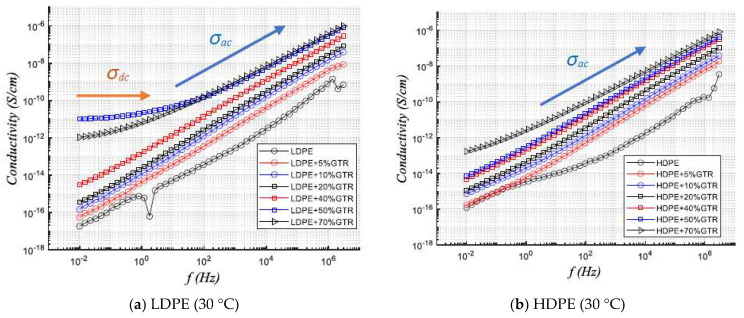
Frequency dependency response of conductivity at 30 °C temperature for (**a**) LDPE/GTR compounds and (**b**) HDPE/GTR compounds.

It should be noted that the phenomena of electrical conduction in these materials are well explained by the theory of conduction called Hopping conduction mechanisms, which has been already introduced and is manifested where there is a change in slope in the conductivity curve (Figure 3a) (i.e., where the AC (σ_ac_) conductivity begins from this slope change, which is called hopping frequency can be modified from the rise of temperatures to higher frequency ranges). At higher frequencies, the charge carriers have more potential for movement and therefore AC conductivity will be dominant [24,25]. In addition, it must be considered that the phenomena of interfacial polarization caused by the MWS (Maxwell–Wagner–Sillar) effect, very remarkable in heterogeneous samples [26,27,28], and which contribute to modifying the conductivity of DC and AC in a substantial, is seen in Figure 3a. As for a maximum of heterogeneity of the system, in the samples LDPE/50%GTR the maximum of the DC conductivity in the system conformed by the compounds LDPE/GTR. This is since the phenomena of intra-chain polarization, i.e., between the main polymer chain and the branched polymer chains, have dielectric repercussions in the conductive behavior of the samples. This is not so in the compounds formed by HDPE/GTR where there is only inter-chain polarization. Therefore the polarization does not increase so much, and it is remarkable therefore that the content of the Carbon Black present in the GTR particles, as a conductive part, increases the conductive behavior.

The test results reflect significant differences in dielectric behavior and results between LDPE matrix and HDPE matrix compounds. In Figure 3a, it is seen for LDPE + GTR compounds how the DC component of the conductivity (*σ_dc_*) is predominant for high concentrations of the GTR reinforcement used (50–70% GTR), in these samples is checked how to arrive at a Hopping frequency or regime change frequency (DC→AC), so, there is a phase change, at a frequency of approximately 10^0^ and 5 × 10^0^ Hz, respectively, and from this frequency value, the behavior of the LDPE samples, dominated by AC conductivity, is observed. The DC conductivity in polymeric compounds follows the following expression [29,30]:

(3) $$\sigma_{dc}=k{\left(v_{f}+v_{fc}\right)}^{t}$$

where k and *t* are constants that depend on the nature of the material, temperature, and the type of reinforcement, on the other hand, *v_fc_* is the critical amount of material (50–70%) and *v_f_* is the percentage of GTR (reinforcement) to use of the 5% to 70% GTR.

Checking Figure 3b, the samples behave very similarly in HDPE compounds, with a dominant AC conductivity phase (σ_ac_), it is remarkable that the sample increases in conductivity as GTR is incorporated into its matrix, up to almost two orders of magnitude in HDPE compounds where 70% GTR is present. This is explained very clearly due to the significant presence of Carbon Black (30%) in the tire particles that have been introduced into the compounds.

**Figure 4:**
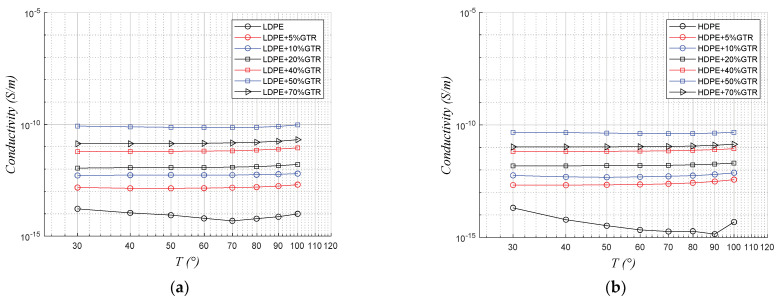
Thermal response of the conductivity with a temperature range of 30 to 100 °C, at 50 Hz, of (**a**) LDPE/GTR compounds and (**b**) HDPE/GTR compounds.

Figure 4 shows the stability of the conductivity with increasing temperature. In the compounds of both LDPE/GTR and HDPE/GTR, there are almost no significant changes, for the frequency that analyzes of 50 Hz, this is since for this frequency the polarization phenomena that mainly affect dipolar structures such as molecules, the changes in conductivity are not very relevant. On the other hand, it is observed how the composition of the samples develop great changes in the conductivity due to the presence of GTR, we observe how the most conductive compounds are those of 50% GTR and not those of 70%, this could be contradictory since with the presence of Carbon Black in this GTR should not be so, but the phenomenon of interfacial polarization, contributes to the increase of conductivity, and that is why in the compounds of 50% of GTR the compounds, for both HDPE and LDPE, they are the most conductive, and the least conductive are the clean compounds without any additives in their matrix.

### 3.3. Permittivity Analysis

**Figure 5:**
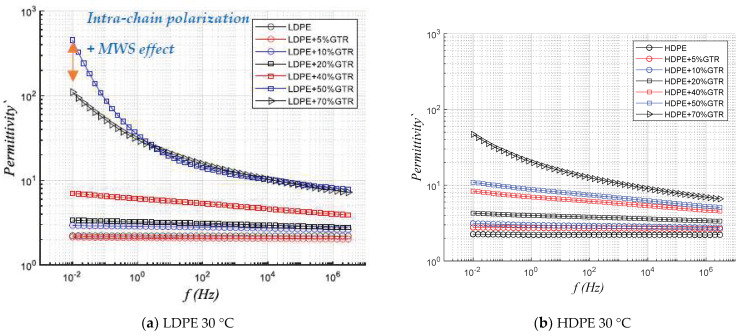
Frequency dependence response of real permittivity, at 30 °C of (**a**) LDPE/GTR compounds and (**b**) HDPE/GTR compounds.

Permittivity describes how the material is affected by the application of an electric field. According to Figure 5, the real permittivity, (ԑ′) and the imaginary permittivity (ԑ″) draw two unequal behaviors (LDPE/GTR). The interfacial polarization due to the MWS effect affects the inter-chain structures combined with intrachain, and there is more affectation in LDPE compounds than HDPE; in HDPE/GTR compounds it is seen that the compounds with the permittivity are the compounds of 70% GTR (Figure 5a). This represents an important difference and leads us to deduce that the effect of interfacial polarization due to the presence of GTR particles in the compounds has a relevant and remarkable impact on branched and low-density compounds such as LDPE. The branching of the LDPE/GTR compounds and the intra-chain polarization, combined with the MWS phenomenon, already introduced, represents the most important reason which explains that the compounds LDPE/GTR 50% are those that have more permittivity unlike the compounds studied of HDPE/GTR where those that present more permittivity are the HDPE/70%GTR. The remarkable behavior of the MWS phenomenon for HDPE is irrelevant, and the behavioral changes are mainly explained by the increase in the presence of Carbon Black in the analyzed HDPE compounds.

**Figure 6:**
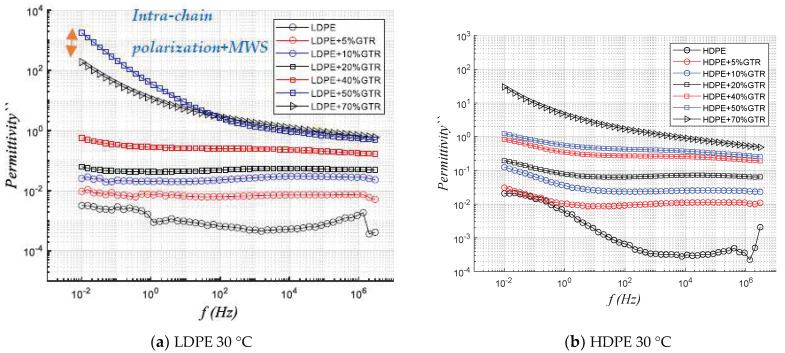
Frequency dependence response of imaginary permittivity, at 30 °C of (**a**) LDPE/GTR compounds and (**b**) HDPE/GTR compounds.

Figure 6 shows that at low GTR concentrations the behavior between HDPE and LDPE composites is very similar, but for high GTR concentrations (50–70%) the behavior differs: For LDPE+50/70%GTR compounds and low frequencies the values are very high (ԑ′: 10^2^–10^3^), and decrease by 10^1^ from 10^4^ Hertz, making it an application material as a capacitive dielectric [31,32].

**Figure 7:**
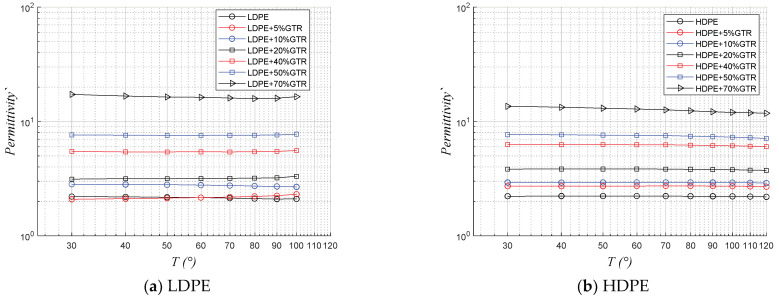
Thermal response of real permittivity (ԑ‱) with a temperature range of 30 to 100 °C, at 50Hz, of (**a**) LDPE/GTR compounds and (**b**) HDPE/GTR compounds.

**Figure 8:**
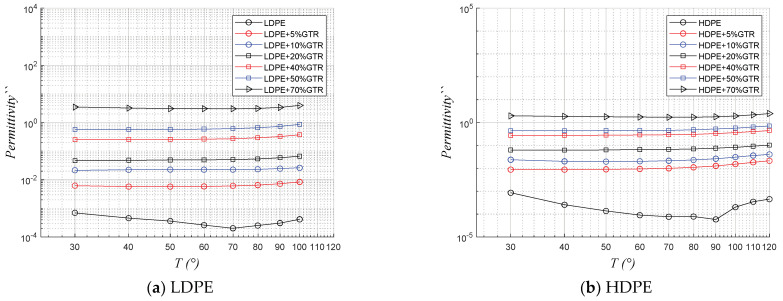
Thermal response of imaginary permittivity (ԑ″) with a temperature range of 30 to 100 °C, at 50 Hz, of (**a**) LDPE/GTR compounds and (**b**) HDPE/GTR compounds.

In Figure 7 and Figure 8, which describe the behavior of both real and imaginary permittivity, respectively, with the evolution of temperature (30 °C to 100 °C), at 50 Hz, so the results show little variability with temperature, a trend similar to that observed with conductivity. However, in this sense, it should also be noted that for these frequencies the compounds with more GTR are those with more permittivity, as the presence of GTR and carbon black has a decisive impact in increasing this at 50 Hz. It is worth noting that the behavior in this case of the compounds LDPE/GTR and HDPE/GTR are similar.

### 3.4. Electrical Modulus 

**Figure 9:**
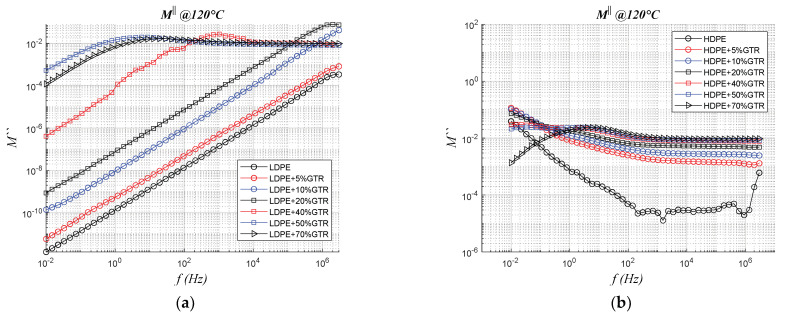

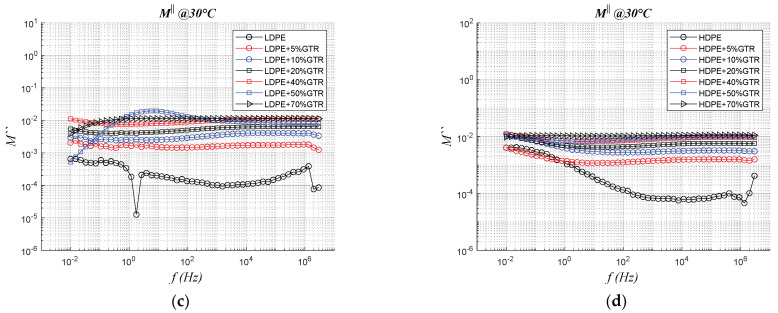
Electrical Imaginary Modulus (M″) on frequency dependence for (**a**) LDPE/GTR at 120 °C; (**b**) HDPE/GTR at 120 °C; (**c**) LDPE/GTR at 30 °C; and (**d**) HDPE/GTR at 30 °C.

The results are shown below using the formalism of the electric module. This expression is used to see much better dielectric relaxation effects that can be masked by other phenomena. Analyzing Figure 9 it is very clear the phenomenon of interfacial polarization caused by heterogeneity in samples such as those analyzed from the presence of two or more phases in a material (matrix + reinforcement), as is the case of the samples analyzed (LDPE+GTR, and HDPE+GTR which are analyzed in Figure 9). From the observation and analysis of Figure 9, it is observed, thanks to the analysis provided by the electric module, that the LDPE polymer, branched and low density is more affected by the presence of GTR in its matrix. This can also be seen in Figure 9c, where we see that the dielectric relaxation at frequencies close to 10^0^–10^1^ Hz is prominent for LDPE-50% GTR compounds. This is again explained due to interfacial polarization phenomena, unlike HDPE/GTR compounds were the most prominent modulus relaxation is for compounds with more GTR amounts in their matrix (HDPE/70%GTR).

**Figure 10:**
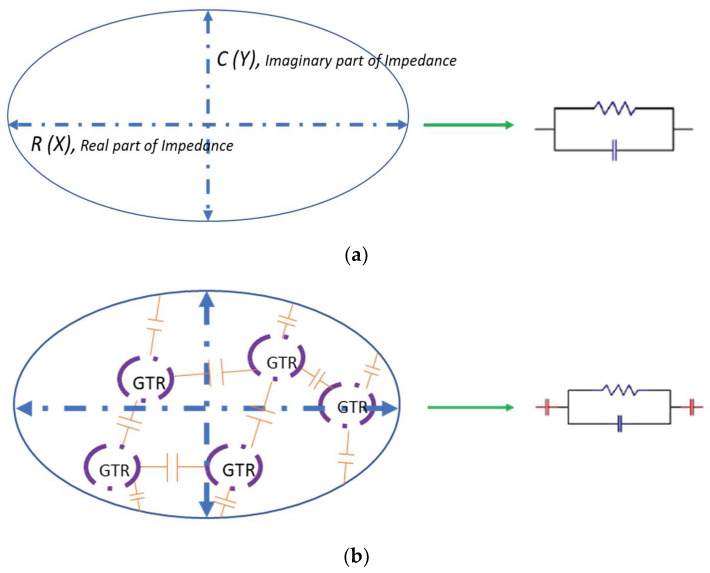
Schematic representation of the dielectric structure and effects-model: (**a**) of LDPE–HDPE neat and (**b**) LDPE composites with GTR reinforcement.

In Figure 10 we see a representation of the dielectric model circuit of the polymers analyzed without GTR particles (13a), and with GTR particles (13b). In this second situation the presence of GTR reproduces a capacitive type of dielectric behavior, assimilated to an R-C circuit, which results in an interfacial polarization, this phenomenon is called Maxwell-Wagner-Sillar (MWS).

### 3.5. Argand Diagrams

**Figure 11:**
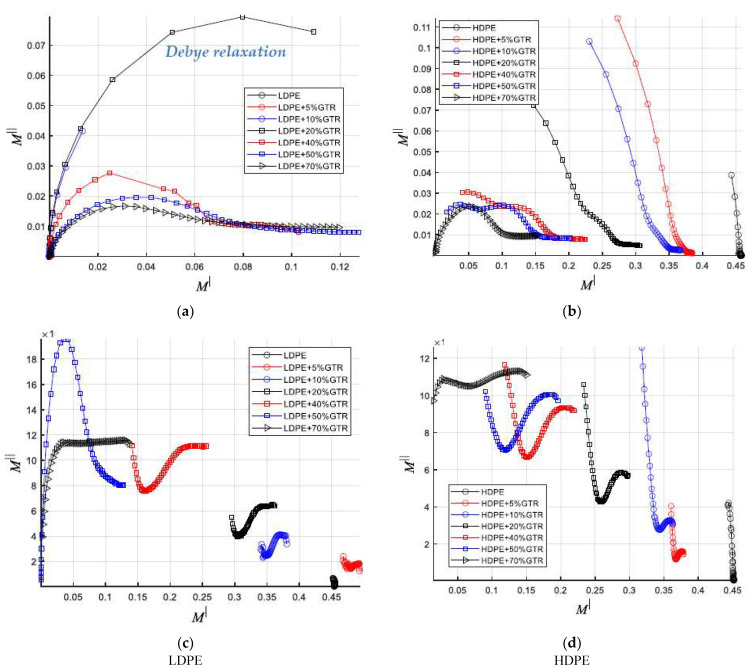
Argand diagram: comparison Figure M″-M′ of (**a**) LDPE/GTR at 120 °C, (**b**) HDPE/GTR at 120 °C, (**c**) LDPE/GTR at 30 °C, and (**d**) HDPE/GTR at 30 °C.

Figure 11 shows the representation of the impedance in a vector diagram or Argand diagram, using the Electrical Module (M′, M″). The impedance of the material can be considered as a vector quantity and represented in the Argand diagram through a point. We see that as we increase the GTR content in the compounds the dielectric behavior is assimilated more and more as the GTR content increases, although it presents differences, in form and behavior in the electrical module for the two types of LDPE/GTR and HDPE/GTR analyzed compounds. Figure 11a shows a Coelho-type relaxation that resembles a Debye relaxation for neat LDPE polymer, this behavior changes with to non-Debye dielectric behavior with GTR addition. Coelho’s [33,34] space charge contribution theory is based on the macroscopic dipole concept. When an electric field is applied to the sample, the mobile carriers move towards the opposite sign electrode, leaving opposite sign carriers next to the other electrode, resulting once the equilibrium is reached with the following charge distribution. This distribution constitutes a macro dipole that would oscillate in an alternating field with the frequency of the field, causing a relaxation process that affects the heat of the permittivity of the medium. For the development of the model, it is assumed that the processes that regulate charge transport are ohmic conduction and diffusion caused by the concentration gradient of the carriers. 

**Figure 12:**
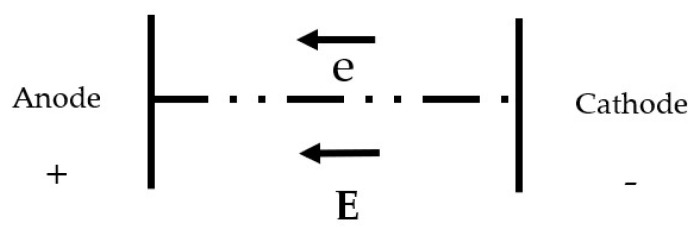
Schematic representation of the charge density distribution in a continuous field that represents the macroscopic dipole in the Coelho model, where ***e*** is the circulation of electrons and ***E*** electric field.

Coelho has shown that the Argand diagram corresponding to the relaxation of space charge is very similar to the Cole-Cole semicircle [35,36,37], the behavior of a Coelho-type relaxation, such as the one observed in Figure 11a for the purely LDPE samples, is assimilated to a model where the charges pass directly from the cathode to the anode, as well as the electric field, as seen in Figure 12.

## 4. Conclusions

In conclusion, HDPE/GTR and LDPE/GTR compounds can create materials that may be suitable as dielectric insulators [38], as they do not lose their insulating properties for low GTR concentrations (<20% of GTR). Also, the analyzed compounds can be good insulators or capacitive dielectrics with permittivity values that are high for these applications for high concentrations of GTR (50–70% GTR). LDPE/50%GTR has especially high values of dielectric constant (ԑ′ = 567) at frequency values of 10^−2^ Hz, and it allows one to obtain a remarkable result as a capacitive dielectric application. Thus, the clear application of this research is that LDPE+50GTR% compounds are applicable to capacitor insulation, increasing the potential of the capacitor. As a resume there are two suitable types of applications from the present research. LDPE and HDPE with low GTR amounts (10%) are suitable as low requirements insulators for applications like electrical protective work shoes, spacers for electric wires, and universal joints for electrical wires (these applications are of low dielectric requirements). On the other hand, capacitive insulators for LDPE with higher GTR amounts (50–70%) are suitable composites as dielectric capacitors, according to the permittivity values checked.

Structurally the morphology of the samples analyzed with SEM show GTR particles poorly integrated into the LDPE and HDPE matrix. LDPE/GTR compounds are more affected by interfacial polarization phenomena (the appearance of this phenomenon is explained by the presence of MWS relaxations) and that occur in heterogeneous compounds, with two or more different phases in a compound. The fact that it manifests itself very clearly is explained by the intra-chain polarization, as it causes the maximization of MWS phenomenon or interfacial polarization. HDPE/GTR (HDPE is high density and unbranched) and its contributions to conductivity and permittivity are explained by the presence of Carbon Black in the GTR particles in the matrix. In summary, the intra-chains of the LDPE branched polymer reinforce the MWS phenomenon and interfacial polarization phenomena, favoring the movement of electrical charges within the compound and accentuating conductivity and permittivity, especially at low frequencies.

## Figures and Tables

**Table 1:** Physical Characteristics LDPE-HDPE.

Feature	LDPE	HDPE
Density, g/cm^3^	0.92–0.93	0.95–0.96
Tensile strength × 1000 PSI	8	5.4
Elongation at break, %	720	700
Crystallinity, %	65	95
Maximum use temperature, °C	82–100	80–120
Flow index g/10 min	1	3
